# Evaluation and validation of methodologies for the extraction of per- and polyfluoroalkyl substances (PFASs) in serum of birds and mammals

**DOI:** 10.1007/s00216-022-03962-3

**Published:** 2022-02-19

**Authors:** Drew Szabo, Jaye Marchiandi, Mark P. Green, Raoul A. Mulder, Bradley O. Clarke

**Affiliations:** 1 Australian Laboratory for Emerging Contaminants, School of Chemistry, University of Melbourne, Victoria, 3010 Australia; 2 School of BioSciences, University of Melbourne, Victoria, 3010 Australia

**Keywords:** Per- and polyfluoroalkyl substance (PFAS), Blood, Avian, Human, Horse, Sample preparation, Solid-phase extraction (SPE)

## Abstract

Advances in analytical techniques have allowed greater detection of environmental contaminants from small volumes of sample. Four methodologies were evaluated for the extraction of 53 per- and polyfluoroalkyl substances (PFASs) from eight classes in 200 µL of avian and mammal serum. Spiked serums at four concentrations (0, 0.5, 5.0 and 25 ng mL^−1^) were prepared by protein precipitation (PPT), enhanced matrix removal (EMR), weak anion exchange (WAX), and hydrophilic-lipophilic balance (HLB) solid-phase extraction cartridges. The extract from each methodology was analysed by high-performance liquid chromatography coupled with tandem mass spectrometry (HPLC–MS/MS), and concentrations were compared with known concentrations in the spiked media. EMR performed the best overall, with 40 of 53 compounds effectively recovered at 5 ng mL^−1^. Furthermore, EMR was effective overall at concentrations ranging from 0.5 to 25 ng mL^−1^ for 39 out of 53. Similarly, PPT was effective for 35 of 53 compounds at all spiked serum concentrations. There was a negative correlation between internal standard recovery for compounds with increasing octanol–water coefficients (*K*_ow_) for WAX (*R* =  − 0.65, *p* = 0.0043) and HLB (*R* =  − 0.62, *p* = 0.0077) extractions, indicating methanol may not be a suitable solvent for long-chain PFAS extraction from protein-rich tissues. EMR and PPT represent fast and effective methodologies for the extraction of PFASs from low volumes of serum which allows greater accuracy and precision that can be applied to future human and wildlife biomonitoring programmes.

**Supplementary Information:**

The online version contains supplementary material available at 10.1007/s00216-022-03962-3.

## Introduction

The development of analytical techniques to quantitatively measure per- and polyfluoroalkyl substances (PFASs) in serum is essential due to the strong bioaccumulation potential and associated negative impacts of PFASs in humans and wildlife. Currently, there are 4730 registered CAS numbers of substances that can be defined as PFASs [1]. PFASs of variable carbon chain lengths can be categorised into discrete classes based on their functional group, such as perfluoroalkanecarboxylic acids (PFCAs) and perfluoroalkanesulfonic acids (PFSAs), among others [2]. PFASs continue to be used for a variety of industrial and commercial purposes [3], such as aqueous film forming foam (AFFF), Scotchgard™ and Teflon™ despite evidence demonstrating their persistence, environmental accumulation and adverse health effects [4]. PFASs enter the environment by intentional [5] and accidental [6] discharge of PFAS-containing products and can then bioaccumulate in plants and animals exposed to contaminated soil and/or water [7, 8]. Therefore, there is an increasing demand to improve the measurement of PFASs in biological matrices to include a wide range of chemistries more easily and affordably.

Whilst PFCAs and PFSAs such as perfluorooctanesulfonic acid (PFOS, C_8_HF_17_O_3_S), perfluorooctanoic acid (PFOA, C_8_HF_15_O_2_) and perfluorohexanesulfonic acid (PFHxS, C_6_HF_13_O_3_S) are the most prevalent PFASs monitored in environmental matrices [9], industrial uses have shifted toward short-chain PFCAs (C_n<7_), and PFSAs (C_n<8_) as well as perfluorinated ether substances (PFES) of lower molecular weight [10]. Hexafluoropropylene oxide-dimer acid (HPFO-DA, or “GenX”, C_6_HF_11_O_3_), perfluoro-3-methoxypropanoic acid (PFMPA, C_4_HF_7_O_3_), perfluoro-4-methoxybutanoic acid (PFMBA, C_5_HF_9_O_3_), perfluoro(2-ethoxyethane)sulfonic acid (PFEESA, C_4_HF_9_O_4_S) and nonafluoro-3,6-dioxaheptanoic acid (NFDHA, C_5_HF_9_O_4_) are examples of short-chain PFES now included in routine monitoring of drinking water in the USA [11]. These substitutes share similar properties to their legacy counterparts owing to their analogous chemical structure, but their environmental occurrence in biological tissues from humans and wildlife has not yet been adequately investigated [12].

The distribution of PFASs throughout body tissue in wildlife is species specific and can be impacted by environmental and physiological changes [13]. Monitoring blood and blood products is commonly employed as a reliable and accurate indicator for total PFAS body burden [14]. PFASs are found in relatively high concentrations in blood and blood products from fish [15], birds [16] and humans [17]. Though, other perfused tissues, such as liver, lung [18] and brain [19], will also accumulate PFASs in significant concentrations. Nevertheless, blood is a preferable biological matrix to study as it can be collected without destroying the subject or without intrusive surgery and allows for longitudinal studies on specific individuals from a population over time [20]. Due to the strong protein binding affinity of PFASs for serum albumins [21], they are found in greater concentrations in serum or plasma compared to whole blood [22]. Therefore, serum is a more useful biomonitor for PFAS exposure in humans and wildlife.

Generally, the sample volume needed to detect PFASs in trace concentrations has decreased from 5 mL [23] to 0.1 mL [24], reflecting the need to reduce the cost and storage per sample and the impact to the subject, whilst maintaining relevant method reporting limits for human and wildlife biomonitoring (0.1 to 1 ng mL^−1^). The total number of PFASs monitored has increased from PFCAs and PFOS-related substances [25] to include PFASs with many chain lengths and functional groups due to their increased prevalence in the environment [26]. PFAS extraction is similar to those currently used for the extraction of other organic compounds from serum which include solid-phase extraction (SPE), protein precipitation (PPT) and ion pairing (Table S1). Keller et al. [27] previously validated six standardised methods for the extraction of 13 of the most commonly measured PFASs in an interlaboratory study. Kaiser et al. [26] then expanded the number of PFASs to include 61 compounds from six functional groups, by solid-phase extraction and ion pairing methodologies. Given the complexity of the serum matrix and potential for interferences in the confirmation of ions [28], research is needed to validate the extraction of a wider set of PFASs to reflect the growing list of compounds that are now routinely monitored in the environment.

In the last decade, exposure to PFASs has been investigated in several human and wildlife biomonitoring studies across multiple countries. Typical background concentrations of PFASs in serum and plasma from human and wildlife could be expected to range from 0.1 to 100 ng mL^−1^. These studies have reported mean levels of PFASs in the general population (e.g. 2.4 ng [PFOS] mL^−1^, 2.1 ng [PFOA] mL^−1^) [29], the regionally exposed people (e.g. 98.1 ng [PFOS] mL^−1^, 98.2 ng [PFOA] mL^−1^) [30] and the occupationally exposed people (e.g. 5624 ng [PFOS] mL^−1^, 1052 ng [PFOA] mL^−1^) [31]. Whilst recent study of three species of bird from France found elevated concentrations of PFASs in serum, with means ranging from 0.2 ng [PFHpS] g^−1^ ww to 60.23 ng [PFOS] g^−1^ ww [32], and mean concentrations of PFASs in captive tigers ranging from 0.05 ng [PFTrDA] mL^−1^ to 2.04 ng [PFOA] mL^−1^ [33]. Exposure to PFASs creates the potential for a suite of adverse health effects in humans [8, 34, 35], and wildlife [36].

This study aims to provide a fast, simple and robust extraction method for the trace quantification of 53 PFASs, from eight functional chemistry classes, in relatively small volumes (< 1 mL) of serum. This is achieved by comparing the extraction efficiency and instrument response for each of the compounds and classes from a range of methodologies. Avian and mammal serum (including human serum) was used to validate existing and novel extraction methods for a wide range of PFAS compounds and the application of the evaluated methodologies will allow clear and reproducible results from human and wildlife biomonitoring programmes.

## Materials and methods

### Chemicals and materials

Standards of 53 native PFASs, including 18 mass-labelled internal standards, were obtained from Wellington Laboratories (Ontario, Canada). Compounds from eight classes were included: eleven perfluoroalkanecarboxylic acids (PFCAs), nine perfluoroalkanesulfonic acids (PFSAs), three perfluoroalkanephosphinic acids (PFPiAs), three fluorotelomercarboxylic acids (FTCAs), four fluorotelomersulfonic acids (FTSAs), eleven perfluoroalkanesulfonyl fluorides (PASFs), four disubstituted fluorotelomer phosphate diesters (diPAPs) and eight perfluoroalkyl ether substances (PFESs). A complete list of studied compounds and their CAS numbers can be found in supplementary information (Table S2). A mix of native PFASs was prepared to 100 ng/mL in methanol.

Hypergrade acetonitrile (> 99.8%; 75–05-8), hypergrade methanol, (> 99.9%; 67–56-1), formic acid (> 99%, 64–18-6) and ammonium acetate (> 99.99%; 631–61-8) were purchased from Sigma-Aldrich (New South Wales, Australia). Type I ultrapure water was obtained from reverse osmosis water coupled with Milli-Q Reference A + system (18.2 Ω, < 5 ppm TOC, Merck, New South Wales, Australia). Chicken serum (16,110–082) and heat-inactivated horse serum (26,050–070) of New Zealand origin were purchased from Thermo Fisher (Victoria, Australia) and human serum was donated from a participating investigator.

Captiva enhanced matrix removal-lipid cartridges (EMR-lipid, 1 cc, 40 mg) were obtained from Agilent Technologies (Delaware, USA). Weak anion exchange cartridges (WAX, 3 cc, 150 mg) and hydrophilic-lipophilic balance cartridges (HLB, 3 cc, 200 mg) were obtained from Waters Corporation (New South Wales, Australia).

### Overview

Four methodologies for the extraction of 53 PFASs from eight classes were evaluated by comparing measured concentration of serum spiked with the known concentrations for each compound. The measured concentrations at four spiked levels were compared statistically to the known concentration to evaluate the efficiency of the method at a range of concentrations. (1) Protein precipitation (PPT) is conducted by the addition of acetonitrile to serum, which results in the formation of a white precipitate, which is then separated by centrifugation or filtration. (2) Enhanced matrix removal (EMR) is a proprietary sorbent developed by Agilent (Delaware, USA), designed to reduce matrix effects by trapping lipids by size exclusion and hydrophobic interactions in a slightly acidic solution (with the addition of 1% formic acid) and allowing aliphatic compounds, such as PFASs, to pass through. The use of acetonitrile also has the effect of precipitating proteins on the cartridge which are then filtered from the extract—further reducing matrix interferences. (3) Weak anion exchange (WAX) and (4) hydrophilic-lipophilic balance (HLB) are sorbents developed by Waters Corporation (New South Wales, Australia), each designed to retain compounds like PFASs by hydrophobic and electrostatic interactions in aqueous solutions. A strong solvent (such as methanol) is then used to elute PFASs from the cartridges for analysis.

### Extraction evaluation

**Fig. 1:**
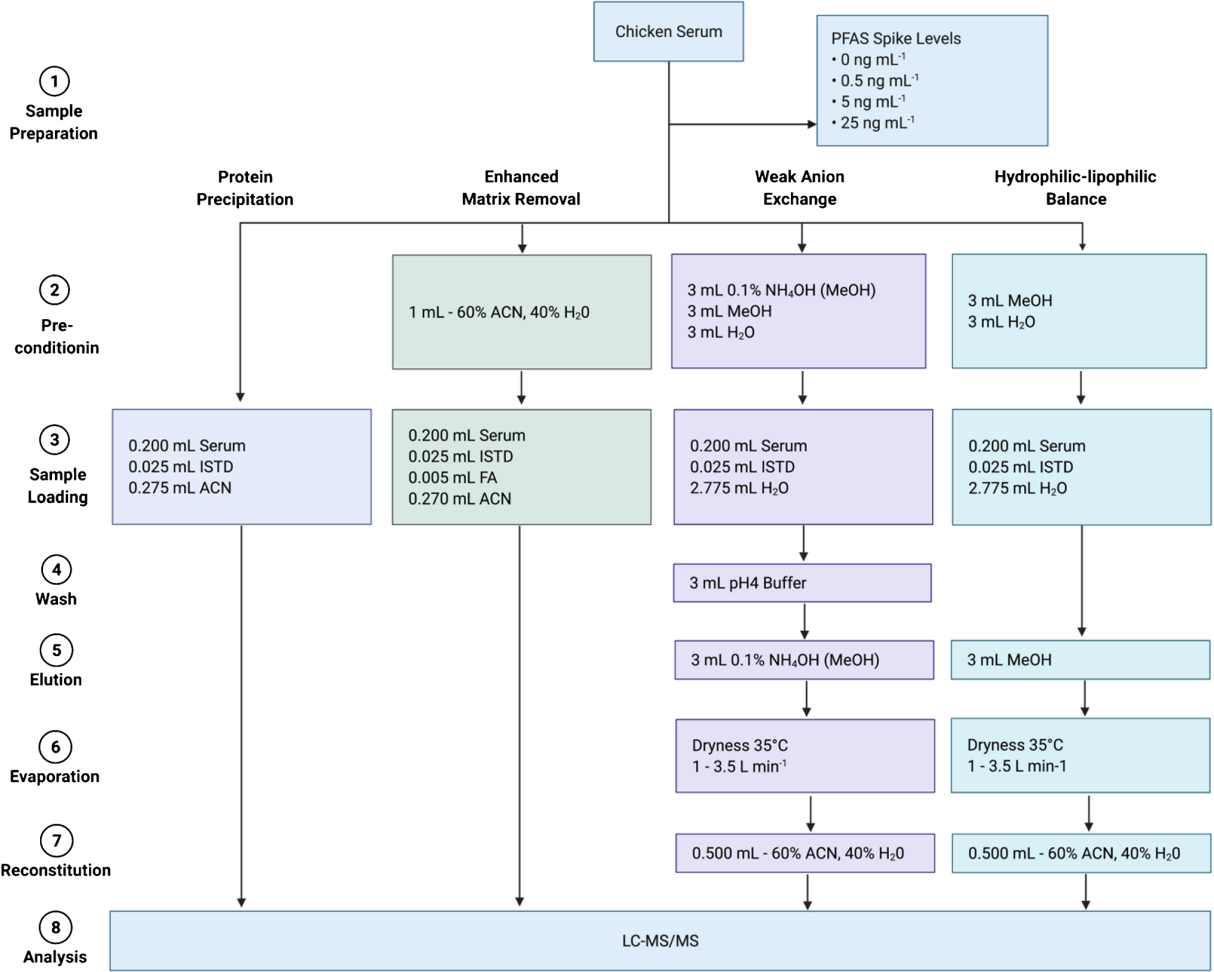
Overview of the four methodologies used for the extraction of PFASs from serum. ACN, acetonitrile; ISTD, internal standard; FA, formic acid; LC–MS/MS, liquid chromatography-tandem mass spectrometry; MeOH, methanol

Chicken serum was used to evaluate and compare the four extraction methodologies (Fig. 1). The extraction of PFASs from chicken serum was conducted at four concentrations: 0 ng mL^−1^ (control), 0.5 ng mL^−1^, 5 ng mL^−1^ and 25 ng mL^−1^ (treatment), which includes the range of commonly detected PFASs from humans and wildlife exposed to background levels and contaminated land and water. A mixed solution of native PFASs in methanol (0.5 mL) was added gravimetrically to reach target concentrations for treatment groups and the methanol was allowed to evaporate to dryness at room temperature. Then, frozen chicken serum was allowed to thaw at 4 °C; then, 10 mL was transferred to the four polypropylene centrifuge tubes each. The serum was vortexed for 30 min and allowed to equilibrate for 24 h at 4 °C.

Equal volumes of spiked serum were used for each extraction methodology and the final extract volume was kept consistent at approximately 0.2 mL serum to 0.3 mL acetonitrile to reliably compare the extraction efficiencies and matrix interferences. In methods requiring the use of methanol (WAX and HLB), the solvent was evaporated and reconstituted in the final extract solution of 60% acetonitrile.

### Extraction validation

The methodology which performed the best for the extraction of PFASs in chicken serum was applied to samples of serum from human and horse samples. The extraction of PFASs from human and horse serums was conducted at four concentrations: 0 ng mL^−1^ (control), 0.5 ng mL^−1^, 5 ng mL^−1^ and 25 ng mL^−1^ (treatment). A mixed solution of native PFASs in methanol (0.200 mL) was added gravimetrically to reach target concentrations for treatment groups and the methanol was allowed to evaporate to dryness at room temperature. Frozen human and horse serums were allowed to thaw at 4 °C; then, 2 mL was transferred to the four polypropylene centrifuge tubes each. The serum was vortexed for 30 min and allowed to equilibrate for 24 h at 4 °C. Triplicate serum samples of each spiked concentration (0.200 mL) were extracted using EMR cartridges on a positive-pressure displacement manifold, as detailed above.

### LC–MS/MS analysis

Sample analysis was performed on an Agilent Technologies 1290 Infinity II liquid chromatography (LC) coupled with Agilent 6495C tandem mass spectrometer (MS/MS) with Agilent Jet Stream negative electrospray ionisation (AJS ESI-) [37]. Separation was achieved on a Zorbax eclipse plus RRHD C18 column (3.0 × 50 mm, 1.8 µm, Agilent Technologies, USA) with a C18 guard column attached. Gradient elution (14 min) using 2-mM ammonium acetate in ultrapure water (A) and MeOH (B) at 400 µL min^−1^ was used. Source conditions for the mass spectrometer are as follows: drying gas = 250 °C at 11 L min^−1^, sheath gas = 375 °C at 11 L min^−1^, nebuliser pressure = 25 psi, capillary and nozzle voltage =  − 2500 V and − 1500 V, and high- and low-pressure RF (iFunnel) =  − 90 V and − 60 V. The m/z transition with the highest intensity was used for quantitation (complete MS/MS parameters listed in Table S2). Linear calibration curves for all compounds to the average of the triplicate injections of ten individual levels from 0.05 to 50 ng mL^−1^ (*R*^2^ > 0.99) and with an accuracy of ± 30% were achieved in MeOH. 4:2 FTSA, 6:2 FTSA, 8:2 FTSA and 10:2 FTSA were linear across eight calibration points (*R*^2^ > 0.99) from 0.05 to 10 ng mL^−1^.

### Quality assurance and quality control

Internal standard recovery was determined by comparing the response of each sample to the average response of the calibration curve. The method reporting limit (MRL) was defined by the lowest calibration concentration with a signal-to-noise ratio (*S*/*N*) greater than 10:1 or three times the concentration in the blank calibration solution, whichever is greater. Samples with *S*/*N* between 3 and 10 were defined as less than the method reporting limit (< MRL) and responses with *S*/*N* less than 3:1 were considered non-detected (n.d.). The qualifier ratio (where two transitions were available) was set to ± 20% of the median response ratio of the calibration curve. Acceptable retention times (RT) were defined as 5% relative to the internal standard response of the calibration standards. If criteria for qualifier ratio or RT were not met, the sample was designated < MRL.

Several PFASs were detected in the blank chicken serum matrix above the method reporting limits. Background concentrations of PFASs detected in serum were relatively consistent within triplicate samples and between extraction methodologies. PFBA was detected in serum extracted by EMR, WAX and HLB methodologies ranging in concentration between 0.31 and 0.90 ng mL^−1^. FOSAA was detected in chicken serum extracted by WAX (average: 0.24 ng mL^−1^) and HLB (average: 0.14 ng mL^−1^) methodologies. PFPeA (average: 0.62 ng mL^−1^) and PFHxA (average: 0.38 ng mL^−1^) were detected in chicken serum extracted by EMR. The average concentrations of PFAS detected triplicate blank samples were subtracted from subsequent treatment groups to obtain a blank-corrected recovery.

### Statistical analyses

Concentration data were acquired and quantitated using Agilent MassHunter Workstation v10.1 and Quantitative Analysis v10.1 respectively. The log octanol–water partition coefficient (log *K*_oc_) for compounds was predicted by the EPI Suite v4.11 KOWWIN™ package [38] where mass-labelled internal standards were assumed to have the same value as the native compound. The KOWWIN™ model performed well in predicting log *K*_ow_ against measured values from the literature [39]. Descriptive statistics, statistical tests and data visualisation were performed with R v4.0.2 using RStudio v1.2.5019 and the following packages: tidyverse v1.3.0.9000 [40], rstatix v0.060 [41] and RColorBrewer v1.1–2 [42]. Internal standard-corrected recoveries were calculated as a percentage of measured concentration to spiked concentration and were assessed by deviation from the expected value. Furthermore, internal standard-corrected instrument responses were plotted against internal standard-corrected concentration for each compound and analysed by linear regression. The estimates for the coefficient were compared statistically to the calibration curve (target concentration) by ANOVA with post hoc Tukey’s pairwise test to determine the accuracy of each method.

$${H}_{0}:{{ B}_{\mathrm{Cal}}= B}_{\mathrm{PPT}}= {B}_{\mathrm{EMR}}= {B}_{\mathrm{WAX}}= {B}_{\mathrm{HLB}}$$

where *B* is the regression coefficient for the calibration curve (Cal), protein precipitation (PPT), enhanced matrix removal (EMR), weak anion exchange (WAX) and hydrophilic-lipophilic balance (HLB) respectively. PFASs were measured in blank serum; the application of the standard addition method is used to measure recovery without blank subtraction. The linearity (*R*^2^) from 0.5 to 25 ng mL^−1^ for each compound is reported here but must be interpreted objectively as there are only four points each.

## Results and discussion

### Method performance (mass recoveries)

**Fig. 2:**
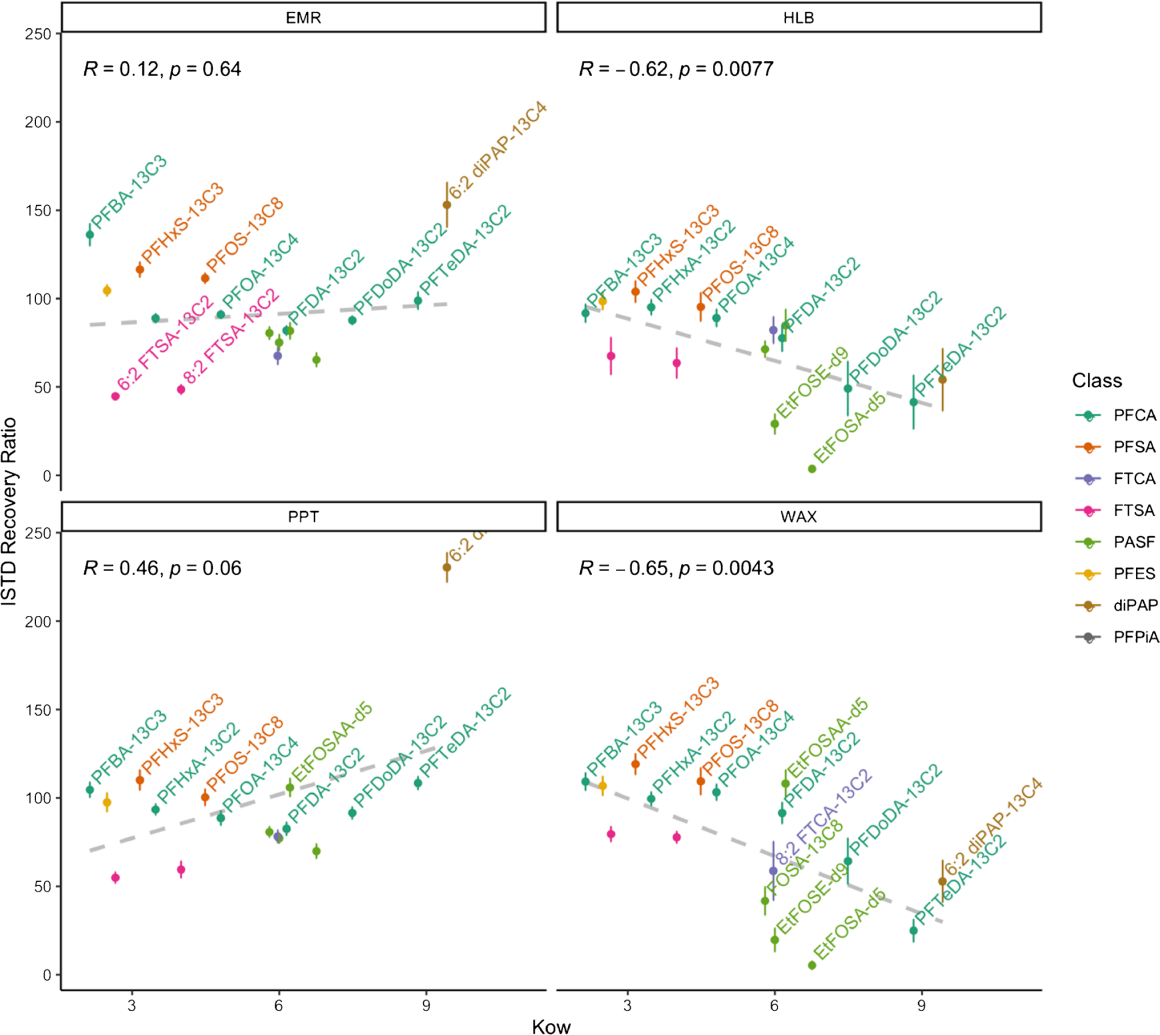
Relationship between the recovery of mass-labelled internal standards and the predicted KOWWIN™ octanol–water partition coefficient [38] for each extraction methodology

The evaluation of four methods for the extraction of PFASs from serum is based on the internal standard-corrected concentrations of each compound with its mass-labelled analogue. The recoveries of 18 mass-labelled internal PFAS standards from six classes were compared to the average response from 10 calibration solutions (injected in triplicate), each spiked with 5 ng mL^−1^ for each compound. The internal standard recoveries for protein precipitation (PPT) and enhanced matrix removal (EMR) were performed within acceptable ranges for most compounds. Average recoveries for PPT ranged from 55 ± 3% to 123 ± 5%, except for 6:2 diPAP-^13^C_4_ which was 230 ± 8%, and EMR ranged from 45 ± 2% to 153 ± 13% for all compounds (Fig. 2). The weak anion exchange (WAX) and hydrophilic-lipophilic balance (HLB) extraction methodologies had greater variability in recovery, where PFTeDA-^13^C_2_, EtFOSA-d_5_ and EtFOSE-d_9_ each had recoveries far below 50%—indicating that the estimation for concentrations of long-chain PFCAs and some PASFs may be negatively impacted by these methodologies. There is a moderate negative correlation for the recovery of compounds with higher octanol–water partition coefficient for the WAX and HLB methodologies (Fig. 2). Chelcea et al. [39] noted that the strong affinity of PFASs to proteins can lead to a discrepancy between *K*_ow_ and bioconcentration factors in biological matrices. The increased hydrophobicity of these compounds and their affinity for proteins in the serum [21] may be stronger than the methanol used to elute the compounds from the WAX and HLB cartridges and further investigation to the strength of solvent used for elution is required. Therefore, the EMR and PPT extraction methodologies for PFASs are preferred, as there is no clear trend for decreasing internal standard recovery with the octanol–water partition coefficient.

The use of mass-labelled internal standards for the quantification of any compound by mass spectrometry is the “gold standard” as you can more accurately estimate the efficiency of the extraction method by the addition of a known concentration of compounds with identical chemical properties [43]. Comparing the response from the mass-labelled internal standard from the calibration levels and samples is a useful tool to measure extraction efficiencies. However, due to the expense of isotopically labelled standards, many researchers use the response from another compound for quantification. This can result in biased recoveries and measurements of concentrations, due to the sorption coefficients of compounds with different carbon chain lengths. It may be impractical to purchase isotopically labelled standards for each native congener; however, the selection of standards should reflect the complete range of functional group chemistry and chain lengths to prevent reporting biased results. For example, if the response from PFOA-^13^C_4_ (C_8_ PFCA) was applied to PFTrDA (C_14_ PFCA), the internal standard-corrected concentration would appear much lower compared with using PFTrDA-^13^C_2_ as the internal standard. The internal standard recoveries should be reported as part of the quality assurance criteria (even if they are single-point calibration), along with the standard that is used to measure the concentrations for each compound.

### Internal standard-corrected recoveries (5 ng/mL)

The internal standard-corrected recovery of 53 PFASs was evaluated from triplicate serum samples spiked at 5 ng mL^−1^ (*n* = 3) by four extraction methodologies, equal to the concentration of 18 mass-labelled PFASs (5 ng mL^−1^). Overall, recovery for PPT ranged from 5.3 ± 0.24% to 181 ± 2.9%, EMR ranged in recovery from 0.01 ± 0.20% to 131 ± 4.9%, WAX ranged in recovery from 1.6 ± 0.43%, and HLB ranged in recovery from 8.3 ± 0.26% to 170 ± 4.5%.

**Fig. 3:**
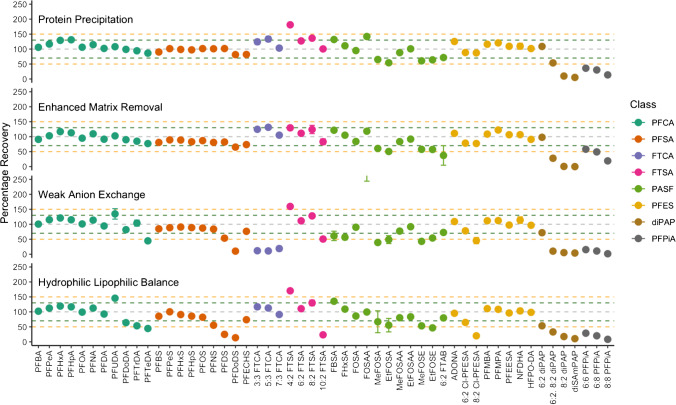
Plot of the average internal standard-corrected recoveries and standard deviation of 53 PFASs from eight classes extracted by protein precipitation, enhanced matrix removal, weak anion exchange and hydrophilic-lipophilic balance. Green and orange lines represent the 70–130% and 50–150% recovery ranges respectively

EMR performed within an acceptable range for the most compounds (40/53) at 5 ng mL^−1^ (Fig. 3). All eleven PFCAs and nine PFSAs, the most commonly measured compounds, were all recovered effectively between 70 and 130%. Eleven PASFs were also effectively recovered, with the exception of 6:2 FTAB (37 ± 32%); all eight PFESs, three FTCAs and four FTSAs were also recovered within ranges of 50 to 150%. Of the diPAPs, only 6:2 diPAP was recovered effectively, whilst 6:2, 8:2 diPAP; 8:2 diPAP; and diSAmPAP were recovered < 28%. Similarly, all three PFPiAs were not effectively recovered by EMR, with recovery < 58%.

EMR sorbent is designed to exclude biological matrix interferences, such as phospholipids, by size exclusion and hydrophobic interactions. diPAPs have a bis(polyfluoroalkane) phosphate ester and PFPiAs have bis(perfluoroalkane) phosphate functional groups, which are structurally similar to phospholipids which have a phosphate ester group between two aliphatic carbon chains. The chemistry of the phosphate functional group in diPAPs and PFPiAs, or the very long chain length, may be negatively affecting the extraction of these compounds by EMR extraction.

### Effects of concentration on recovery

The average internal standard-corrected recovery of PFASs from three treatment levels (0.5, 5.0 and 25 ng mL^−1^) and seven classes was successful in at least one methodology (Table 1). The perfluorophosphinic acids (PFPiAs) were not able to be recovered consistently by the tested methodologies. To the authors’ knowledge, PFPiAs are not routinely monitored in abiotic or biotic matrices [12] and are typically not detected in drinking water [44], surface water [45], wastewater or soil [46]. Whilst PFPiAs are offered for a range of consumer and industrial applications including wetting agents in household cleaning products [47], there is little information on the toxicology of PFASs with this functional group to make an assessment about their associated exposure risks.

**Table 1:** Summary of the average internal standard-corrected recoveries of PFASs from each extraction methodology at 0.5, 5 and 25 ng mL^−1^ and results of 95% family-wise ANOVA and post hoc Tukey’s analysis against the expected concentration. *p*-values in bold highlight the accuracy for the recovery of compounds at all treatment levels that do not have a difference to the expected concentration on average

	**PP**		**EMR**		**WAX**		**HLB**		**ANOVA**
	**Recovery (%)**	**Tukey’s *p*-value**	**Recovery (%)**	**Tukey’s *p*-value**	**Recovery (%)**	**Tukey’s *p*-value**	**Recovery (%)**	**Tukey’s *p*-value**	
*PFCA*									
PFBA	111 ± 5.7	0.0138	81 ± 20.5	**0.4788**	104 ± 5.6	**0.1966**	108 ± 8.6	0.0011	*F* = 4.63, *p* = 0.0010
PFPeA	128 ± 11.2	0.0021	99 ± 13.7	0.0391	128 ± 11.6	0.0015	124 ± 9.3	0.0008	*F* = 7.01, *p* = 0.0000
PFHxA	138 ± 8.2	0.0012	113 ± 11.7	0.0215	129 ± 5.7	0.0087	127 ± 9.3	0.0054	*F* = 6.99, *p* = 0.0000
PFHpA	140 ± 7.8	0.0001	122 ± 7.8	**0.0873**	122 ± 6.2	0.0324	120 ± 10.4	0.0079	*F* = 6.72, *p* = 0.0000
PFOA	112 ± 5.9	0.0047	101 ± 5.4	**0.9996**	106 ± 4.2	**0.3098**	102 ± 9	**0.0966**	*F* = 4.07, *p* = 0.0026
PFNA	123 ± 8	0.0023	117 ± 5.5	0.0384	119 ± 5.4	0.0213	114 ± 14.3	0.0015	*F* = 5.95, *p* = 0.0001
PFDA	107 ± 6	**0.1699**	95 ± 3.8	**0.8217**	96 ± 3.8	**0.6761**	91 ± 9.8	**0.8775**	*F* = 4.49, *p* = 0.0013
PFUDA	109 ± 6.5	**0.6581**	102 ± 3.8	**0.9879**	135 ± 11.1	0.0028	115 ± 40.2	0.0079	*F* = 5.79, *p* = 0.0002
PFDoDA	103 ± 4.6	**0.9998**	93 ± 3.1	**0.7573**	78 ± 9.8	**0.0507**	52 ± 15.1	0.0006	*F* = 6.23, *p* = 0.0000
PFTrDA	94 ± 3.4	**0.7349**	83 ± 2.6	**0.3374**	86 ± 23.7	**0.0516**	35 ± 20	0.0002	*F* = 5.86, *p* = 0.0001
PFTeDA	84 ± 11.5	**0.3587**	76 ± 9.6	**0.2196**	39 ± 22.7	0.0028	32 ± 10.3	0.0036	*F* = 6.52, *p* = 0.0000
*PFSA*									
PFBS	96 ± 5.8	**0.8365**	86 ± 6.4	0.0136	90 ± 4.8	**0.1044**	95 ± 9.2	**0.6758**	*F* = 5.39, *p* = 0.0003
PFPeS	111 ± 8.6	**0.1677**	96 ± 8.2	**0.9919**	96 ± 6.7	**0.9966**	108 ± 8.2	**0.1078**	*F* = 4.59, *p* = 0.0011
PFHxS	104 ± 5.8	**0.9948**	92 ± 3.3	0.03	95 ± 4.1	**0.3202**	94 ± 9.4	**0.9839**	*F* = 4.49, *p* = 0.0013
PFHpS	102 ± 5.4	**0.9999**	89 ± 6.8	**0.0565**	96 ± 6.7	**0.5925**	88 ± 8.8	**0.6847**	*F* = 5.15, *p* = 0.0004
PFOS	104 ± 5.3	**0.9999**	88 ± 6.1	**0.1023**	91 ± 3.8	**0.2097**	83 ± 6.7	**0.1009**	*F* = 5.05, *p* = 0.0005
PFNS	103 ± 5.8	**0.9784**	83 ± 4.4	**0.2082**	79 ± 5.4	**0.0604**	52 ± 8.4	0.0003	*F* = 6.55, *p* = 0.0000
PFDS	99 ± 6.5	**0.8947**	83 ± 5.7	**0.439**	49 ± 7.8	0.0095	22 ± 3.5	0.0003	*F* = 6.91, *p* = 0.0000
PFDoDS	78 ± 19.5	**0.2705**	66 ± 18.6	**0.1422**	10 ± 3.9	0.0028	12 ± 3.7	0.0034	*F* = 6.78, *p* = 0.0000
PFECHS	93 ± 9.4	**0.9616**	84 ± 10.1	**0.576**	85 ± 8.2	**0.7089**	80 ± 5.6	**0.6704**	*F* = 4.56, *p* = 0.0011
*FTCA*									
3:3 FTCA	93 ± 51.7	**0.8034**	94 ± 50.5	**0.7975**	9 ± 7.6	0.0001	87 ± 50.1	**0.9339**	*F* = 6.94, *p* = 0.0000
5:3 FTCA	109 ± 48	**0.3519**	106 ± 48.7	**0.4027**	10 ± 8.8	0.0011	92 ± 41.3	**0.4027**	*F* = 6.66, *p* = 0.0000
7:3 FTCA	92 ± 28.1	**0.9895**	90 ± 31.8	**0.9901**	20 ± 12.5	0	81 ± 25.1	**0.9934**	*F* = 7.22, *p* = 0.0000
*FTSA*									
4:2 FTSA	177 ± 8.7	0.0184	129 ± 4.1	**0.35**	154 ± 10.7	**0.1456**	167 ± 12.4	**0.0708**	*F* = 6.77, *p* = 0.0000
6:2 FTSA	132 ± 11.4	**0.1511**	109 ± 4	**0.579**	109 ± 4.5	**0.5358**	108 ± 11.2	**0.3898**	*F* = 6.30, *p* = 0.0000
8:2 FTSA	129 ± 15.3	**0.989**	115 ± 18.3	**1**	115 ± 12.3	**0.9994**	110 ± 21.6	**0.9999**	*F* = 4.50, *p* = 0.0013
10:2 FTSA	98 ± 11.2	**0.9817**	82 ± 9.2	**0.6165**	41 ± 8.9	0.0018	20 ± 4.5	0	*F* = 7.36, *p* = 0.0000
*PASF*									
FBSA	98 ± 60.6	**0.0594**	91 ± 56.8	**0.2268**	42 ± 27.4	**0.1684**	103 ± 66.1	0.0108	*F* = 6.48, *p* = 0.0000
FHxSA	82 ± 53.2	**0.4529**	76 ± 49.1	**0.9137**	43 ± 25	0.0016	81 ± 54.3	**0.3818**	*F* = 6.67, *p* = 0.0000
FOSA	78 ± 33.8	**0.9979**	70 ± 28.2	**0.1636**	72 ± 27	**0.0609**	68 ± 31.3	**0.1014**	*F* = 6.29, *p* = 0.0000
FOSAA	150 ± 10.2	**0.1288**	125 ± 7.6	**0.6254**	287 ± 68.1	0	102 ± 7	**0.9934**	*F* = 9.65, *p* = 0.0000
EtFOSA	50 ± 39.4	**0.122**	48 ± 35.1	0.0301	44 ± 35.8	0.0059	74 ± 70.9	0.0029	*F* = 8.57, *p* = 0.0000
EtFOSAA	105 ± 5.7	**0.9807**	96 ± 3	**0.9218**	91 ± 2.8	**0.4218**	80 ± 7	**0.0827**	*F* = 4.87, *p* = 0.0007
EtFOSE	55 ± 33.9	**0.3453**	51 ± 31.2	**0.1994**	42 ± 24.4	0.0064	37 ± 21.1	0.0014	*F* = 7.65, *p* = 0.0000
MeFOSA	56 ± 44	**0.5699**	56 ± 44.9	**0.7217**	39 ± 25.1	0	47 ± 40.3	0.0056	*F* = 7.79, *p* = 0.0000
MeFOSAA	97 ± 7.9	**0.9989**	93 ± 9	**0.9467**	86 ± 13.8	**0.3962**	80 ± 4.6	**0.4329**	*F* = 4.49, *p* = 0.0013
MeFOSE	46 ± 29.3	**0.0846**	46 ± 28.8	**0.0836**	32 ± 19.3	0.0019	36 ± 25.4	0.0058	*F* = 7.43, *p* = 0.0000
6:2 FTAB	48 ± 40	**0.867**	51 ± 33.9	**0.3521**	70 ± 37.1	**0.3096**	70 ± 31	**0.0513**	*F* = 5.82, *p* = 0.0001
*PFES*									
ADONA	135 ± 7.9	0	118 ± 5.4	0.0334	116 ± 5.8	**0.0656**	100 ± 8.1	**0.3539**	*F* = 6.45, *p* = 0.0000
HFPO-DA	109 ± 7.5	**0.4274**	95 ± 4.1	**0.6179**	102 ± 5	**0.9999**	108 ± 8.8	**0.3739**	*F* = 2.72, *p* = 0.0262
6:2 Cl-PFESA	93 ± 4.9	**0.867**	82 ± 3.1	**0.3521**	81 ± 3.6	**0.3096**	67 ± 5.4	**0.0513**	*F* = 5.55, *p* = 0.0002
8:2 Cl-PFESA	91 ± 5.2	**0.9701**	81 ± 4.3	**0.718**	43 ± 7.3	0.0215	23 ± 3.9	0.0016	*F* = 6.63, *p* = 0.0000
NFDHA	109 ± 43.2	0.0015	115 ± 15.1	0.0088	120 ± 13.3	0.0195	111 ± 9.5	**0.0837**	*F* = 5.27, *p* = 0.0004
PFEESA	119 ± 8.8	0.0009	113 ± 4.8	0.0451	102 ± 5.1	**0.8487**	104 ± 7.3	**0.3525**	*F* = 4.99, *p* = 0.0006
PFMBA	127 ± 10.2	0.0009	116 ± 6.9	**0.0662**	121 ± 8.5	0.0081	120 ± 9	0.0026	*F* = 6.18, *p* = 0.0000
PFMPA	132 ± 11.1	0.0011	132 ± 7.8	0.0006	122 ± 8.2	0.0161	118 ± 9	0.0189	*F* = 6.52, *p* = 0.0000
*diPAP*									
6:2 diPAP	104 ± 8.7	**0.9999**	96 ± 7.4	**0.9899**	64 ± 14.6	0.0242	40 ± 14.6	0.0014	*F* = 6.01, *p* = 0.0001
6:2, 8:2 diPAP	52 ± 15.9	0.0345	30 ± 12.6	0.0065	17 ± 6.5	0.0047	27 ± 9.1	0.0195	*F* = 7.21, *p* = 0.0000
8:2 diPAP	7 ± 2.1	0.0072	1 ± 0.3	0.0042	8 ± 4.6	0.0113	15 ± 6.5	0.0205	*F* = 7.59, *p* = 0.0000
diSAmPAP	3 ± 2.2	0.0117	0 ± 1.2	0.0089	5 ± 2.8	0.0149	8 ± 5.2	0.021	*F* = 7.37, *p* = 0.0000
*PFPiA*									
6:6 PFPiA	34 ± 9	0.0239	53 ± 14.3	**0.2057**	16 ± 2.4	0.0011	20 ± 14.1	0.0055	*F* = 7.60, *p* = 0.0000
6:8 PFPiA	25 ± 12.8	0.0152	41 ± 18.2	**0.1036**	8 ± 7.1	0.0011	15 ± 11.9	0.0036	*F* = 7.80, *p* = 0.0000
8:8 PFPiA	12 ± 8.9	0.0099	18 ± 11.4	0.0205	2 ± 2.3	0.002	8 ± 6.3	0.0052	*F* = 7.87, *p* = 0.0000

Perfluoroalkylcarboxylic acids (PFCAs) were recovered from PPT (84 ± 15% to 140 ± 8%), EMR (76 ± 10% to 122 ± 8%), WAX (39 ± 23% to 135 ± 11%) and HLB (32 ± 10% to 127 ± 9%) methodologies on average across three concentrations. EMR performed the best on average for PFCA extraction with all compounds recovered within ± 30% of the spiked concentration for each level. Furthermore, the regression coefficients for eight out of eleven PFCAs were not significantly different from the expected concentrations, indicating good recovery over a range of concentrations (0.5 to 25 ng mL^−1^). PPT also performed well for all compounds, except for PFHpA (140 ± 8%) and PFHxA (138 ± 8%) which had slight enhancement. The regression coefficients for compounds extracted by PPT indicate good agreement for C_n>9_ PFCAs with the expected concentration. Recovery of PFASs with WAX and HLB cartridges did not perform within 70–130% for compounds with increasing chain lengths. The recovery of PFTeDA by WAX extraction was 39 ± 23% and recoveries of PFDoDA, PFTrDA and PFTeDA by HLB extraction were 52 ± 15%, 35 ± 20% and 32 ± 10% respectively. Analysis of the regression coefficients for recovery of PFASs by WAX and HLB indicates that few compounds were effectively extracted across the treatment range.

Perfluoroalkylsulfonic acids (PFSAs) were recovered from PPT (78 ± 20% to 111 ± 9%), EMR (66 ± 19% to 96 ± 8%), WAX (10 ± 4% to 95 ± 7%) and HLB (12 ± 4% to 108 ± 8%) methodologies on average across three concentrations. PPT performed the best on average for PFSA extraction with all compounds recovered within ± 30% of the spiked concentration for each level and the regression coefficients for each compound did not differ significantly from the expected concentration. EMR performed well for all PFSAs, except for PFDoDS (66 ± 19%) which had its recovery slightly suppressed. Recovery of PFSAs was acceptable for WAX and HLB extractions, except for PFDS and PFDoDS. Recoveries of PFDS and PFDoDS for WAX were 49 ± 8% and 10 ± 4% respectively, and for HLB, recoveries were 22 ± 4% and 12 ± 4% respectively. Similar to the recovery of long-chain PFCAs, the recovery of long-chain PFSAs was significantly suppressed compared to the expected concentration.

Fluorotelomercarboxylic acids (n:3 FTCAs) were recovered from PPT (92 ± 28% to 109 ± 48%), EMR (90 ± 32% to 106 ± 49%), WAX (9 ± 8% to 20 ± 13%) and HLB (81 ± 25% to 92 ± 41%) methodologies on average across three concentrations. Extraction of FTCAs by PPT, EMR and HLB methodologies performed equally well, each with recoveries ± 30% and no observable difference in the regression coefficient of the expected concentration. WAX extraction did not perform within expected ranges for recovery and the regression coefficient was significantly different to the spiked concentration range.

Fluorotelomersulfonic acids (n:2 FTSAs) were recovered from PPT (98 ± 11% to 177 ± 9%), EMR (82 ± 9% to 129 ± 4%), WAX (41 ± 9% to 154 ± 11%) and HLB (20 ± 5% to 167 ± 12%) methodologies on average across three concentrations. Extraction of FTSAs by EMR performed well for each compound, with recoveries within ± 30% and no observable difference in the regression coefficient compared with the expected concentrations. PPT performed well for most FTSAs, except for 4:2 FTSA (177 ± 9%), which was significantly different to the expected concentrations. WAX and HLB had similar recovery of FTSAs, with increased recovery of 4:2 FTSA (154 ± 11% and 167 ± 12% respectively), and reduced recovery of 10:2 FTSA (41 ± 9% and 20 ± 5% respectively). The regression coefficients for 4:2 FTSA were not significantly different from the expected concentration range, indicating there may have been background contamination that way below the method reporting limit, which inflated the average recovery.

Perfluoroalkylsulfonyl fluorides (PASFs) were recovered from PPT (50 ± 39% to 150 ± 10%), EMR (48 ± 35% to 125 ± 8%), WAX (39 ± 25% to 287 ± 68%) and HLB (36 ± 25% to 103 ± 66%) methodologies on average across three concentrations. Average recoveries for EMR performed better on average for the extraction of most PASFs, with EtFOSA (48 ± 35%), EtFOSE (51 ± 31%), MeFOSA (56 ± 45%), MeFOSA (46 ± 29%) and 6:2 FTAB (51 ± 34%) falling outside the acceptable recovery range. The low average recovery for these compounds is driven by a low recovery at concentrations ≤ 5 ng mL^−1^ (Supplementary Data), as recovery at 25 ng mL^−1^ was 86 ± 4%, 84 ± 3%, 105 ± 5%, 71 ± 2% and 53 ± 2% respectively. Similarly, the average recoveries for these four compounds extracted by PPT were below 70% due to incomplete recovery at concentrations ≤ 5 ng mL^−1^ (Supplementary Data). Extraction of PASFs with WAX only resulted in acceptable recovery for EtFOSAA (91 ± 3%), FOSA (72 ± 27%) and MeFOSAA (86 ± 14%) which each had agreeable regression coefficients compared with the expected concentration. Extraction of PASFs with EMR resulted in acceptable recoveries of EtFOSAA (80 ± 7%), FBSA (103 ± 66%), FhxSA (81 ± 54%), FOSAA (102 ± 7%) and MeFOSAA (80 ± 5%), also with agreeable regression coefficients compared with the expected concentrations.

Perfluoroether substances (PFESs) were recovered from PPT (48 ± 40% to 135 ± 8%), EMR (51 ± 34% to 132 ± 8%), WAX (43 ± 7% to 122 ± 8%) and HLB (23 ± 4% to 120 ± 9%) methodologies on average across three concentrations. PPT and EMR had acceptable recoveries for all compounds within ± 30% (Supplementary Data). WAX extraction was effective in extracting all compounds, except for 8:2 Cl-PFESA which had recoveries below 70% for all concentration levels. Similarly, HLB was not effective in extracting both 6:2 Cl-PFESA and 8:2 Cl-PFESA at all concentration levels but was more effective for all other PFES, as the regression coefficient for many compounds was not significantly different to the expected concentrations.

Disubstituted fluorotelomerphosphate diesters (diPAPs) were recovered from PPT (3 ± 2% to 104 ± 9%), EMR (0 ± 1% to 96 ± 7%), WAX (5 ± 3% to 64 ± 15%) and HLB (8 ± 5% to 40 ± 15%) methodologies on average across three concentrations. Only 6:2 diPAP was recovered effectively by PPT and EMR extraction methodologies with recoveries of 104 ± 9% and 96 ± 7% respectively, each with good agreement with the regression coefficient of the expected concentrations. The application of the internal standard (6:2 diPAP-^13^C_2_) for the quantification of 6:2/8:2 diPAP, 8:2 diPAP and diSAmPAP is likely resulting in the reduced concentration of each of these compounds, as the internal standard response was significantly elevated.

### Considerations for the quantification of PFASs

**Fig. 4:**
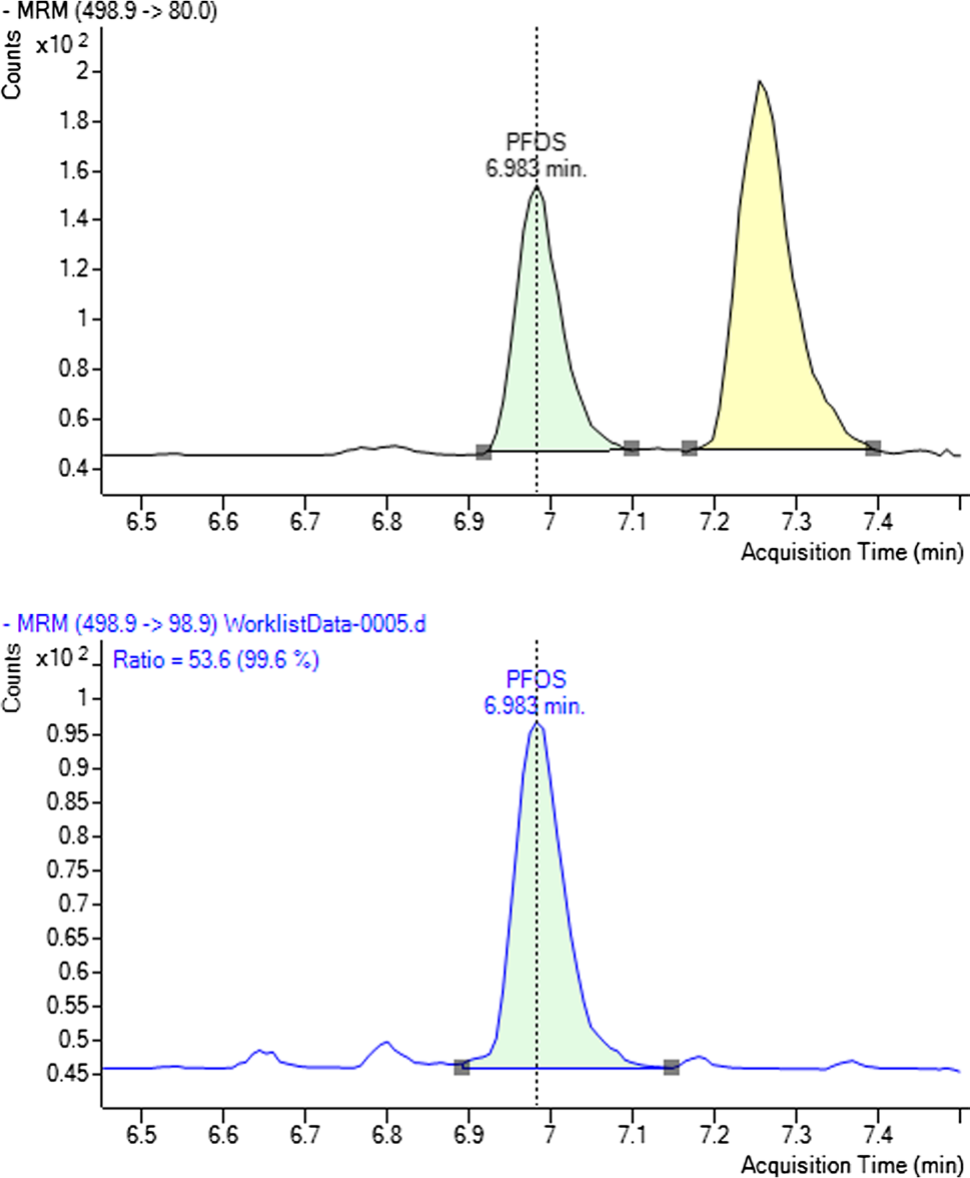
Protein precipitation chromatograms for two transitions of PFOS (m/z = 498.9), top: SO_3_^−^ (m/z = 80.0) and bottom: FSO_3_^−^ (*m*/*z* = 98.9) at 0.5 ng mL^−1^. Note: TDCA peak (m/z = 398.9–80.0) present at RT = 7.23 min

Serum is a complex matrix of biological material, including a range of proteins and lipids of different chemistries. Whilst each extraction methodology aims to reduce the concentrations of each of these materials to exclude them from analysis, the selection of PFASs often includes other organic molecules that can impact the quantification of PFASs by LC–MS/MS, for example taurodeoxycholic acid (TDCA, C_26_H_45_NO_6_S), a bile acid present in serum, that shares a similar retention time, precursor and product ion mass with PFOS, using a C18 column (*m/z* 498.9 → 80.0) [28]. This presents an analytical challenge to prevent the over-quantification of PFOS in biological matrices, as the sulphite fragment (*m/z* 80; SO_3_^−^) is greater in abundance compared to the fluorosulfate fragment (*m/z* 98.9; FSO_3_^−^) [37]. However, as TDCA does not contain any fluorine, the *m/z* 98.9 transition is not present (Fig. 4). Separation of TDCA and PFOS can be achieved on a column using either perfluorooctyl stationary phases [23] or an ion-exchange column [48]. Although TDCA was not removed by any of the extraction protocols used in this study, the slow elution gradient presented in this analytical methodology (*t* = 14.5 min) has achieved separation with a C18 column. Analysis of the *m/z* 80 and 98.9 transitions reveals that PFOS is eluted at *t* = 6.98 min and TDCA is eluted at *t* = 7.23 min where the response returns to baseline between each peak.

As many analytical methodologies strive for shorter run times and faster elution gradients, it is important to ensure that potential interfering compounds, such as TDCA, are separated from PFASs with equal precursor and product ion masses. To this end, the known qualifier ratio of two transitions from the calibration levels must fall within ± 20% of the ratio measured in the sample. Any deviation from the known qualifier ratio may be due to the presence of another compound with a similar retention time and precursor mass. In cases where PFASs have lower precursor mass, and therefore only one positive transition with *m/z* > 100, such as PFBA, PFMBA and PFPeA, the identification and quantification do inherit some risk. As the results for these compounds in this study indicate accurate recoveries, there does not seem to be any interfering matrix components that are suppressing or enhancing the quantification.

### Validation of human and horse serums

EMR performed well as an extraction methodology for PFASs in human and horse serums. All PFCAs, PFSAs and PFESs were effectively extracted from serum at concentrations ranging from 0.5 ng mL^−1^ to 25 ng mL^−1^, except for NFDHA at 0.5 ng mL^−1^ (58 ± 20%). At higher concentrations (5 and 25 ng mL^−1^), FTCAs and FTSAs had slightly elevated recoveries in both horse and human serums. The recoveries of PASFs were more variable, with most compounds effectively recovered at all concentrations and EtFOSA, EtFOSE, MeFOSA and MeFOSE exhibiting very low recovery ≤ 5 ng mL^−1^. Only 6:2 diPAP and 6:6 PFPiA were recovered well from their respective classes. Overall, these results are consistent with results from extraction of PFASs from chicken serum (Table 2).

**Table 2:** Summary of internal standard-corrected recoveries for spiked human and horse serums with EMR extraction methodology

Compound	Horse			Human		Mean ISTD response
	0.5 ng/mL	5 ng/mL	25 ng/mL	5 ng/mL	25 ng/mL	
*PFCA*						
PFBA	108 ± 8	103 ± 12	113 ± 11	121 ± 11	114 ± 13	101 ± 14
PFPeA	109 ± 11	113 ± 2	120 ± 1	125 ± 7	119 ± 4	88 ± 8
PFHxA	109 ± 12	130 ± 3	140 ± 2	139 ± 4	136 ± 6	88 ± 8
PFHpA	94 ± 9	124 ± 2	132 ± 4	128 ± 5	128 ± 4	85 ± 7
PFOA	99 ± 9	114 ± 2	121 ± 4	119 ± 5	119 ± 4	85 ± 7
PFNA	110 ± 12	126 ± 2	133 ± 4	128 ± 5	128 ± 3	75 ± 6
PFDA	98 ± 13	110 ± 2	116 ± 4	113 ± 2	113 ± 4	75 ± 6
PFUDA	113 ± 20	121 ± 2	125 ± 7	123 ± 6	118 ± 5	68 ± 8
PFDoDA	101 ± 13	103 ± 2	109 ± 3	106 ± 5	107 ± 5	68 ± 8
PFTrDA	104 ± 31	103 ± 7	117 ± 25	119 ± 15	112 ± 8	68 ± 16
PFTeDA	98 ± 8	91 ± 3	84 ± 4	101 ± 4	99 ± 6	68 ± 16
*PFSA*						
PFBS	92 ± 11	106 ± 3	110 ± 4	113 ± 3	113 ± 7	120 ± 11
PFPeS	105 ± 12	104 ± 4	114 ± 5	100 ± 4	110 ± 5	114 ± 9
PFHxS	102 ± 20	110 ± 4	116 ± 4	115 ± 4	118 ± 5	114 ± 9
PFHpS	97 ± 4	115 ± 3	119 ± 3	121 ± 2	121 ± 6	99 ± 6
PFECHS	91 ± 7	99 ± 3	104 ± 3	103 ± 3	104 ± 6	99 ± 6
PFOS	98 ± 22	106 ± 5	109 ± 1	114 ± 4	113 ± 7	99 ± 6
PFNS	86 ± 10	108 ± 2	105 ± 2	110 ± 4	107 ± 9	99 ± 6
PFDS	93 ± 6	103 ± 2	103 ± 3	111 ± 4	110 ± 6	99 ± 6
PFDoDS	89 ± 2	91 ± 10	84 ± 12	112 ± 5	114 ± 7	99 ± 6
*FTCA*						
3:3 FTCA	88 ± 22	179 ± 14	182 ± 12	154 ± 7	148 ± 9	51 ± 7
5:3 FTCA	76 ± 14	187 ± 11	194 ± 20	175 ± 11	171 ± 6	51 ± 7
7:3 FTCA	32 ± 31	142 ± 6	149 ± 8	142 ± 6	146 ± 7	51 ± 7
*FTSA*						
4:2 FTSA	294 ± 40	331 ± 21	287 ± 10	135 ± 7	124 ± 6	40 ± 5
6:2 FTSA	127 ± 10	129 ± 7	124 ± 7	131 ± 3	119 ± 5	40 ± 5
8:2 FTSA	112 ± 25	158 ± 6	145 ± 10	158 ± 15	137 ± 4	38 ± 5
10:2 FTSA	64 ± 56	97 ± 3	89 ± 4	117 ± 9	104 ± 4	38 ± 5
*PASF*						
FBSA	20 ± 9	130 ± 7	162 ± 14	139 ± 4	160 ± 11	71 ± 7
FHxSA	21 ± 6	130 ± 6	147 ± 8	140 ± 5	149 ± 10	71 ± 7
FOSA	34 ± 10	109 ± 3	115 ± 3	111 ± 2	115 ± 8	71 ± 7
FOSAA	102 ± 17	120 ± 2	122 ± 2	107 ± 6	106 ± 8	71 ± 7
EtFOSA	0 ± 5	5 ± 1	74 ± 5	6 ± 2	66 ± 3	50 ± 10
EtFOSAA	103 ± 21	106 ± 3	109 ± 2	102 ± 3	101 ± 4	56 ± 9
EtFOSE	2 ± 0	9 ± 1	57 ± 2	10 ± 0	31 ± 2	60 ± 12
MeFOSA	3 ± 4	10 ± 1	96 ± 22	13 ± 0	87 ± 4	50 ± 10
MeFOSAA	110 ± 10	105 ± 5	111 ± 6	99 ± 3	96 ± 4	56 ± 9
MeFOSE	2 ± 6	16 ± 2	58 ± 11	19 ± 2	40 ± 3	60 ± 12
6:2 FTAB	0 ± 0	62 ± 7	61 ± 5	61 ± 6	68 ± 6	99 ± 6
*PFES*						
6:2 Cl-PFESA	91 ± 11	99 ± 2	101 ± 2	102 ± 4	107 ± 6	99 ± 6
8:2 Cl-PFESA	81 ± 12	98 ± 4	104 ± 5	105 ± 5	110 ± 7	99 ± 6
ADONA	119 ± 11	128 ± 1	137 ± 5	133 ± 5	134 ± 4	85 ± 7
HFPO-DA	100 ± 10	110 ± 3	116 ± 4	114 ± 2	114 ± 6	78 ± 7
NFDHA	58 ± 20	83 ± 8	93 ± 2	113 ± 11	105 ± 6	88 ± 8
PFEESA	114 ± 8	122 ± 3	128 ± 0	134 ± 5	131 ± 6	88 ± 8
PFMBA	106 ± 10	114 ± 3	120 ± 2	122 ± 5	118 ± 5	88 ± 8
PFMPA	106 ± 10	112 ± 2	116 ± 1	120 ± 4	118 ± 5	88 ± 8
*diPAP*						
6:2 diPAP	98 ± 16	105 ± 2	106 ± 4	106 ± 4	103 ± 3	110 ± 34
6:2, 8:2 diPAP	3 ± 4	4 ± 1	5 ± 2	28 ± 6	23 ± 8	110 ± 34
8:2 diPAP	0 ± 0	0 ± 0	0 ± 0	1 ± 1	1 ± 1	110 ± 34
diSAmPAP	1 ± 3	0 ± 0	0 ± 0	0 ± 0	0 ± 0	110 ± 34
*PFPiA*						
6:6 PFPiA	65 ± 25	84 ± 13	105 ± 39	109 ± 33	91 ± 12	110 ± 34
6:8 PFPiA	35 ± 26	53 ± 14	66 ± 15	89 ± 21	82 ± 10	110 ± 34
8:8 PFPiA	0 ± 0	6 ± 6	11 ± 1	41 ± 11	46 ± 5	110 ± 34

Extraction efficiencies from human serum by EMR are equal to or exceed WAX and HLB methodologies previously reported, especially for long-chain PFCAs and PFSAs [26], likely due to their increased affinity for serum protein. In an interlaboratory study, Keller et al. [27] found increased relative standard deviation (RSD) for long-chain PFCAs (PFDA, PFUDA) potentially due to the lower concentrations in the human serum samples, although the affinity of these compounds for serum protein may also explain the variability in results due to different extraction methodologies used (WAX, HLB, C18, PPT, ion pairing).

## Recommendations and conclusion

The results from this study establish a robust, fast and simple methodology for the extraction of the widest range of PFASs from serum of multiple species compared with those previously published (Table S1). There is evidence that compounds with relatively high *K*_ow_ are not successfully recovered by WAX and HLB cartridges. As these compounds are typically recovered well from other matrices [37], this negative effect is likely due to the strong interactions between the compounds and the serum protein. Elution with a stronger organic solvent may be applied to help the recovery; however, the use of acetonitrile has similar results with this study [26]. Overall, enhanced matrix removal (EMR) was the most successful extraction methodology for chicken, horse and human serums as the majority of compounds ranging in concentration from 0.5 to 25 ng mL^−1^ were recovered within acceptable thresholds. This methodology could be scaled to allow for quantification of higher concentrations (with a greater calibration range), although decreasing the limit of reporting is currently challenging with the volume of serum at 0.2 mL. This methodology is an important contribution to the quantification of PFASs, in small volumes of biological matrices for improved and effective biomonitoring application.

## Supplementary Information

Below is the link to the electronic supplementary material.

Supplementary file1 (DOCX 4.24 MB)
