# Supplementary material for: Evaluation and validation of methodologies for the extraction of per- and polyfluoroalkyl substances (PFASs) in serum of birds and mammals
Source: Anal Bioanal Chem. 2022 Feb 19;414(9):3017–32. doi: 10.1007/s00216-022-03962-3 (PMC8934760; doi:10.1007/s00216-022-03962-3)
Supplement: Supplementary file 1 — Supplementary file1 (DOCX 4.24 MB) [file 216_2022_3962_MOESM1_ESM.docx]

SUPPLEMENTARY INFORMATION

Evaluation and validation of methodologies for the extraction of per- and polyfluoroalkyl substances (PFASs) in serum of birds and mammals

Drew Szabo^1^, Jaye Marchiandi^1^, Mark P. Green^2^, Raoul A. Mulder^2^ & Bradley O. Clarke^1#^

^1^ Australian Laboratory for Emerging Contaminants, School of Chemistry, University of Melbourne, Victoria, Australia 3010.

^2^ School of BioSciences, University of Melbourne, Victoria, Australia 3010.

| Table S1: Comparison of methodologies used to extract PFASs from blood, plasma and serum. | | | | | | |
| --- | --- | --- | --- | --- | --- | --- |
| **Number of PFAS measured** | **^13^C-PFAS_n_** | **Functional Groups** | **Extraction** | **Clean up** | **Sample/matrix volume** | **Reference** |
| 53 | 18 | PFSA, PFCA, FTCA, FTSA, PASF, PFES, diPAP, PFPiA | 1. SPE: WAX, HLB, EMR  2. Protein Precip |  | 0.2 mL | This study |
| 61 | 37 | PFSA, PFCA, FTSA, PASF, PFES, diPAP | 1. SPE: WAX, HLB  2. Ion-pairing | 1. GNPC | 0.5 mL | Kaiser, Aro [1] |
| 51 | 22 | PFCA, PFSA, FTCA,  FTSA, PASF, PFES, diPAP,  PFPiA | 1. Protein Precip |  | 0.2 mL | Da Silva, Ahmadireskety [2] |
| 28 | 23 | PFSA, PFCA, FTSA, PASF, diPAP | 1. Protien Precip | 1. MCX | 0.1 mL | Nakayama, Isobe [3] |
| 6* | 0 | PFSA, PFCA | 1: SPE: Fe_3_O_4_@mSiO_2_-F_17_ |  | 0.1 mL | Liu, Yu [4] |
| 8 - 13 | 3 - 9 | PFSA, PFCA, PASF | 1. SPE: WAX  2. SPE: C18 (on-line)  3. Protein Precip  4. Ion-pairing  5. Ion-pairing  6. SPE: HLB |  | 1. 0.15 – 1.2 g  2. 0.2 mL  3. 0.2 mL  4. 1mL  5. 0.2 – 0.5 g  6. 0.2 mL | Keller, Calafat [5] |
| 28 | 5 | PFSA, PFCA, PASF | 1. SPE: WAX  2. Protein Precip  3. Formic Acid | 3. SPE: WAX | 1 mL  (whole blood) | Yeung, Taniyasu [6] |
| 0* | 3 | PFSA, PFCA | 1. Ion-pairing  2. SPE: C18  3. Protein Precip |  | 0.1 – 1 mL | [7] |
| 11 | 5 | PFSA, PFCA, PASF | 1. Formic Acid | 1. SPE: WAX | 5 mL | Benskin, Bataineh [8] |
| 12 | 2 | PFSA, PFCA, PASF | 1. SPE: C18 |  | 0.75 mL | Kärrman, van Bavel [9] |
| 13* | 1 | PFSA, PFCA, PASF | 1. SPE: HLB |  | 1 mL | Kuklenyik, Reich [10] |
| *PFSA: perfluorosulfonic acid, PFCA: perfluorocarboxylic acid, FTCA: fluorotelomercarboxylic acid, FTSA: fluorotelomersulfonic acid, PASF: perfluroalkylsulfonyl fluoride, PFES: perfluoroether-based substances, diPAP: disubstituted fluorotelomerphosphate diesters, PFPiA: perfluorophosphinic acid, SPE: solid-phase extraction, WAX: weak anion exchange, HLB: hydrophilic-lithophilic balance, EMR: enhanced matrix removal, MCX: mixed-mode cation exchange, MTBE: methyl-tert butyl ether, TBA: tetrabutylammonium, PSA: primary secondary amine, GNPC: graphitised non-porous carbon.*  **Additional ^12^C-PFAS used as internal standard* | | | | | | |

## Methodology

The protein precipitation method was performed by the addition of 0.200 mL spiked chicken serum to a 2 mL polypropylene cryovial with the addition of mass-labelled internal standard (0.025 mL), formic acid (0.005 mL) and hypergrade acetonitrile (0.270 mL). The sample was briefly vortexed (30 sec, 2000 rpm) centrifuged (10 min, 2000 *g*) before the supernatant was transferred to a 0.250 mL polypropylene autosampling vial for analysis.

Three solid-phase extraction methods were performed using the reverse-phase weak anion exchange (WAX), hydrophilic-lipophilic balance (HLB) cartridges, and the normal-phase enhanced matrix removal (EMR) cartridge. WAX cartridges were first pre-conditioned with 3 mL 0.1% ammonium hydroxide in methanol, and then both WAX and HLB cartridges were pre-conditioned with 3 mL methanol and then 3 mL of ultrapure water under vacuum (17 kPa). While the sorbent was still wet, water (2.775 mL) was added to the cartridge and mixed with serum (0.2 mL) and mass-labelled internal standard (0.025 mL), then the cartridge was eluted under vacuum (17 kPa). The WAX cartridge was rinsed with 3 mL pH 4 buffer (sodium acetate/acetic acid). WAX cartridges were then eluted with 3 mL 0.1% ammonium hydroxide in methanol and HLB cartridges were eluted with 3 mL methanol. The eluent from WAX and HLB cartridges were evaporated to dryness and reconstituted to 0.5 mL with 60% acetonitrile in water for analysis. The practice of evaporating samples containing PFASs to dryness at low temperatures (<35 °C) has been validated in previous studies, and has not resulted in volitisation or thermal degredation of selected compounds [11, 12].

The EMR cartridges were pre-conditioned with 1 mL 60% acetonitrile on a positive-pressure displacement manifold (Agilent, USA) at 20 kPa with ultrapure nitrogen. Spiked chicken serum (0.200 mL) was added to the cartridge with mass-labelled internal standard mix (0.025 mL), formic acid (0.005 mL) and hypergrade acetonitrile (0.270 mL). The cartridges were then briefly vortexed (10 sec, 2000 rpm) before the eluent was collected under low-pressure nitrogen (20 kPa) in a 1 mL polypropylene autosampling vial for analysis.

| Table S2: Summary of PFAS names, CAS numbers and mass spectrometry parameters for quantification. | | | | | | | |
| --- | --- | --- | --- | --- | --- | --- | --- |
| **Abbr** | **Compound** | **CAS** | **Prec Ion** | **Prod Ion** | **CE (V)** | **RT (min)** | **Reference** |
| 6:2 diPAP | Bis[2-perfluorohexylethyl] phosphate | 407582-79-0 | 789 | 97 (79) | 28 (68) | 9.43 | 6:2 diPAP-13C4 |
| 8:2 diPAP | Bis[2-perfluorooctylethyl] phosphate | 678-41-1 | 989 | 542.9 (96.9) | 24 (36) | 10.42 | 6:2 diPAP-13C4 |
| 6:2, 8:2 diPAP | 6:2/8:2 Fluorotelomer phosphate diester | 943913-15-3 | 889 | 442.9 (96.9) | 20 (32) | 9.99 | 6:2 diPAP-13C4 |
| diSAmPAP | Bis-[2-N-ethylperfluorooctane-1-sulfonamidoethyl] phosphate | NA | 1203 | 525.9 (168.9) | 48 (72) | 10.8 | 6:2 diPAP-13C4 |
| 8:2 PAP-13C2 |  |  | 545 | 97 | 36 | 7.43 |  |
| 6:2 diPAP-13C4 |  |  | 793 | 444.9 | 20 | 9.43 |  |
| 8:2 diPAP-13C4 |  |  | 993.1 | 544.9 | 20 | 10.52 |  |
| 3:3 FTCA | 3:3 Fluorotelomer carboxylic acid | 356-02-5 | 241 | 176.9 (116.9) | 4 (40) | 4.38 | 8:2 FTCA-13C2 |
| 7:3 FTCA | 7:3 Fluorotelomer carboxylic acid | 812-70-4 | 441 | 337.1 (317) | 8 (16) | 7.15 | 8:2 FTCA-13C2 |
| 5:3 FTCA | 5:3 Fluorotelomer carboxylic acid | 914637-49-3 | 341 | 237 (216.9) | 8 (28) | 5.83 | 8:2 FTCA-13C2 |
| 8:2 FTCA-13C2 |  |  | 479 | 394 | 12 | 7.23 |  |
| 10:2 FTSA | 10:2 Fluorotelomer sulfonic acid | 120226-60-0 | 627 | 606.8 (81) | 32 (40) | 8.7 | 8:2 FTSA-13C2 |
| 6:2 FTSA | 6:2 Fluorotelomer sulfonic acid | 27619-97-2 | 427 | 406.9 (81) | 24 (36) | 6.24 | 6:2 FTSA-13C2 |
| 8:2 FTSA | 8:2 fluorotelomersulfonic acid | 39108-34-4 | 527 | 506.9 (80) | 32 (56) | 7.58 | 8:2 FTSA-13C2 |
| 4:2 FTSA | 4:2 Fluorotelomer sulfonic acid | 757124-72-4 | 327 | 307 (81) | 20 (36) | 4.89 | 6:2 FTSA-13C2 |
| 6:2 FTSA-13C2 |  |  | 428.9 | 408.8 | 24 | 6.24 |  |
| 8:2 FTSA-13C2 |  |  | 529 | 508.8 | 28 | 7.58 |  |
| EtFOSE | 2-N-ethylperfluoro-1-octanesulfonamido-ethanol | 1691-99-2 | 630 | 59.1 | 12 | 9.82 | EtFOSE-d9 |
| MeFOSAA | N-methylperfluoro-1-octanesulfonamidoacetic acid | 2355-31-9 | 570 | 512 (419) | 24 (20) | 7.89 | EtFOSAA-d5 |
| MeFOSE | 2-N-methylperfluoro-1-octanesulfonamido-ethanol | 24448-09-7 | 616 | 59.1 | 12 | 9.47 | EtFOSE-d9 |
| FOSAA | Perfluoro-1-octanesulfonamidoacetic acid | 2806-24-8 | 556 | 497.9 (78) | 32 (52) | 7.53 | EtFOSAA-d5 |
| EtFOSAA | N-ethylperfluoro-1-octanesulfonamidoacetic acid | 2991-50-6 | 584 | 526 (418.9) | 16 (20) | 8.19 | EtFOSAA-d5 |
| FBSA | Perfluoro-1-butylsulfonamide | 30334-69-1 | 298 | 78 | 28 | 5.28 | FOSA-13C8 |
| MeFOSA | N-methylperfluoro-1-octansulfonamide | 31506-32-8 | 512 | 218.9 (168.9) | 24 (28) | 9.46 | EtFOSA-d5 |
| 6:2 FTAB | 6:2 Fluorotelomer sulfonamide betaine | 34455-29-3 | 569.1 | 549 (223.1) | 8 (12) | 7.03 | PFOS-13C4 |
| EtFOSA | N-ethylperfluoro-1-octanesulfonamide | 4151-50-2 | 526 | 219 (168.9) | 28 (28) | 9.83 | EtFOSA-d5 |
| FHxSA | Perfluoro-1-hexanesulfonamide | 41997-13-1 | 397.9 | 78 | 28 | 6.93 | FOSA-13C8 |
| FOSA | Perfluoro-1-octanesulfonamide | 754-91-6 | 497.9 | 78 | 36 | 8.4 | FOSA-13C8 |
| Table S2 cont: Summary of PFAS names, CAS numbers and mass spectrometry parameters for quantification. | | | | | | | |
| **Abbr** | **Compound** | **CAS** | **Prec Ion** | **Prod Ion** | **CE (V)** | **RT (min)** | **Reference** |
| HFPO-DA-13C3 |  |  | 287 | 169 | 4 | 5.13 |  |
| EtFOSAA-d5 |  |  | 589 | 418.8 | 20 | 8.18 |  |
| FOSA-13C8 |  |  | 506 | 78 | 36 | 8.4 |  |
| EtFOSE-d9 |  |  | 639.1 | 59 | 12 | 9.79 |  |
| EtFOSA-d5 |  |  | 531.1 | 168.8 | 28 | 9.81 |  |
| PFUnDA | Perfluoroundecanoic acid | 2058-94-8 | 563 | 518.9 (269) | 8 (16) | 8.18 | PFDoDA-13C2 |
| PFPeA | Perfluoropentanoic acid | 2706-90-3 | 263 | 219 | 4 | 4.35 | PFHxA-13C2 |
| PFHxA | Perfluorohexanoic acid | 307-24-4 | 313 | 269 (119) | 4 (20) | 4.94 | PFHxA-13C2 |
| PFDoDA | Perfluorododecanoic acid | 307-55-1 | 613 | 568.9 (319) | 8 (20) | 8.69 | PFDoDA-13C2 |
| PFOA | Perfluoroocanoic acid | 335-67-1 | 413 | 369 (169) | 8 (16) | 6.28 | PFOA-13C4 |
| PFDA | Perfluorodecanoic acid | 335-76-2 | 512.9 | 468.8 (268.9) | 8 (16) | 7.6 | PFDA-13C2 |
| PFBA | Perfluorobutanoic acid | 375-22-4 | 213 | 169 | 4 | 2.75 | PFBA-13C3 |
| PFHpA | Perfluoroheptanoic acid | 375-85-9 | 363 | 319 (169) | 4 (16) | 5.6 | PFOA-13C4 |
| PFNA | Perfluorononanoic acid | 375-95-1 | 463 | 419 (219) | 8 (16) | 6.96 | PFDA-13C2 |
| PFTeDA | Perfluorotetradecanoic acid | 376-06-7 | 712.9 | 668.9 (168.9) | 8 (28) | 9.53 | PFTeDA-13C2 |
| PFTrDA | Perfluorotridecanoic acid | 72629-94-8 | 663 | 618.9 (168.9) | 8 (28) | 9.13 | PFTeDA-13C2 |
| PFBA-13C3 |  |  | 216 | 171.9 | 4 | 2.74 |  |
| PFHxA-13C2 |  |  | 314.9 | 269.8 | 4 | 4.94 |  |
| PFOA-13C4 |  |  | 417 | 371.9 | 8 | 6.28 |  |
| PFDA-13C2 |  |  | 514.9 | 469.9 | 8 | 7.6 |  |
| PFDoDA-13C2 |  |  | 615 | 569.9 | 8 | 8.69 |  |
| PFTeDA-13C2 |  |  | 715.1 | 669.8 | 8 | 9.53 |  |
| PFEESA | Perfluoro(2-ethoxyethane)sulfonate | 117205-07-9 | 314.9 | 135 (69.1) | 24 (60) | 4.71 | PFBS-13C2 |
| NFDHA | Perfluoro-3,6-dioxaheptanoic acid | 151772-58-6 | 295 | 201 (85) | 0 (28) | 4.84 | PFOA-13C4 |
| PFMPA | Perfluoro-4-oxapentanoic acid | 377-73-1 | 229 | 85 | 8 | 3.72 | PFBA-13C3 |
| HFPO-DA | Perfluoro-2-methyl-3-oxahexanoate (GenX) | 62037-80-3 | 285 | 185 (169) | 16 (4) | 5.13 | HFPO-DA-13C3 |
| 6:2 Cl-PFESA | 9-chlorohexadecafluoro-3-oxanonane-1-sulfonate (F-53B) | 73606-19-6 | 530.9 | 350.8 (204) | 28 (16) | 7.36 | PFOS-13C4 |
| 8:2 Cl-PFESA | 11-chloroeicosafluoro-3-oxaundecane-1-sulfonate (F-53B) | 83329-89-9 | 630.9 | 450.8 (82.9) | 32 (32) | 8.46 | PFOS-13C4 |
| PFMBA | Perfluoro-5-oxahexanoic acid | 863090-89-5 | 279 | 235 (85) | 0 (8) | 4.54 | PFHxA-13C2 |
| ADONA | Dodecafluoro-3H-4,8-dioxanonanoate | 958445-44-8 | 377 | 251 (85) | 8 (36) | 5.67 | PFOA-13C4 |
| PFHxPA | Perfluorohexanephosphonic acid | 40143-76-8 | 398.9 | 79 | 36 | 4.3 | PFHxPA-Cl |
| Table S2 cont: Summary of PFAS names, CAS numbers and mass spectrometry parameters for quantification. | | | | | | | |
| **Abbr** | **Compound** | **CAS** | **Prec Ion** | **Prod Ion** | **CE (V)** | **RT (min)** | **Reference** |
| PFOPA | Perfluorooctanephosphonic acid | 40143-78-0 | 498.9 | 79 | 44 | 5.55 | PFOPA-Cl |
| PFDPA | Perfluorodecylphosphonic acid | 52299-26-0 | 598.9 | 79 | 44 | 6.91 | PFOPA-Cl |
| PFHxPA-Cl |  |  | 414.9 | 79 | 32 | 5.7 |  |
| PFOPA-Cl |  |  | 514.9 | 79 | 40 | 7.12 |  |
| 8:8 PFPiA | Bisheptadecafluorooctylphosphinate | 500776-69-2 | 900.9 | 500.8 (63) | 68 (80) | 10.12 | 6:2 diPAP-13C4 |
| 6:6 PFPiA | Bisperfluorohexylphosphinate | 70609-44-8 | 700.9 | 400.9 (63) | 56 (68) | 8.92 | 6:2 diPAP-13C4 |
| 6:8 PFPiA | Perfluorohexylperfluorooctylphosphinate | NA | 800.9 | 500.9 (400.8) | 60 (68) | 9.6 | 6:2 diPAP-13C4 |
| PFHpS | Perfluoroheptanesulfonic acid | 21934-50-9 | 448.9 | 98.9 (80) | 44 (50) | 6.32 | PFOS-13C4 |
| PFPeS | Perfluoropentanesulfonic acid | 2706-91-4 | 348.9 | 99 (80) | 40 (40) | 5.03 | PFHxS-13C3 |
| PFDS | Perfluorodecanesulfonic acid | 2806-15-7 | 598.9 | 98.9 (80) | 52 (64) | 8.16 | PFOS-13C4 |
| PFBS | Perfluorobutanesulfonic acid | 29420-49-3 | 298.9 | 98.9 (80) | 36 (36) | 4.51 | PFBS-13C2 |
| PFOS | Perfluorooctanesulfonic acid | 4021-47-0 | 498.9 | 98.9 (80) | 48 (49) | 6.98 | PFOS-13C4 |
| PFECHS | Perfluoro-4-ethylcyclohexanesulfonate | 67584-42-3 | 460.93 | 380.8 (98.9) | 28 (32) | 6.22 | PFOS-13C4 |
| PFDoDS | Perfluorododecanesulfonic acid | 79780-39-5 | 698.9 | 98.9 (79.9) | 60 (64) | 9.1 | PFOS-13C4 |
| PFHxS | Perfluorohexanesulfonic acid | 82382-12-5 | 398.9 | 99 (80) | 36 (50) | 5.65 | PFHxS-13C3 |
| PFNS | Perfluorononanesulfonic acid | 98789-57-2 | 548.9 | 98.9 (80) | 52 (52) | 7.59 | PFOS-13C4 |
| PFBS-13C2 |  |  | 302 | 79.9 | 44 | 4.51 |  |
| PFHxS-13C3 |  |  | 402 | 98.9 | 40 | 5.65 |  |
| PFOS-13C4 |  |  | 503 | 99 | 48 | 6.98 |  |
| PFOS-13C8 |  |  | 507 | 98.8 | 48 | 6.98 |  |

## Evaluation of sample preparation

A qualitative evaluation of the removal of background and potentially interfering matrix effects was performed by the analysis of all precursor ions in total ion transmission mode between *m/z* 100 – 1300 (Figure S1). The baseline for each method performed equally from 3.5 – 9 min where the concentration of methanol is increased from 10% to 90%. EMR performed best and WAX and HLB were also effective in reducing the total ion transmission at high organic concentrations at 9 – 12 min. There was more ion transmission for the protein precipitation method at higher organic concentrations. EMR, WAX and HLB are all sorbent-based extraction methods designed to remove a range of background ions from the matrix and clean up the extract, whereas protein precipitation is designed only to remove substances that are precipitated by acetonitrile, allowing the coextraction of matrix components not targeted by this methodology. The total ion chromatogram is representative of all the compounds negatively ionised by the electrospray source, and thereby potentially allowing the suppression of the ionisation of target anions. Although, the presence of these untargeted matrix ions will not generally affect the quantification of target compounds, as the selection of the precursor and product ions (±0.7 amu) by the first and third quadrupole in MRM mode allow high confidence in compound identification.

| 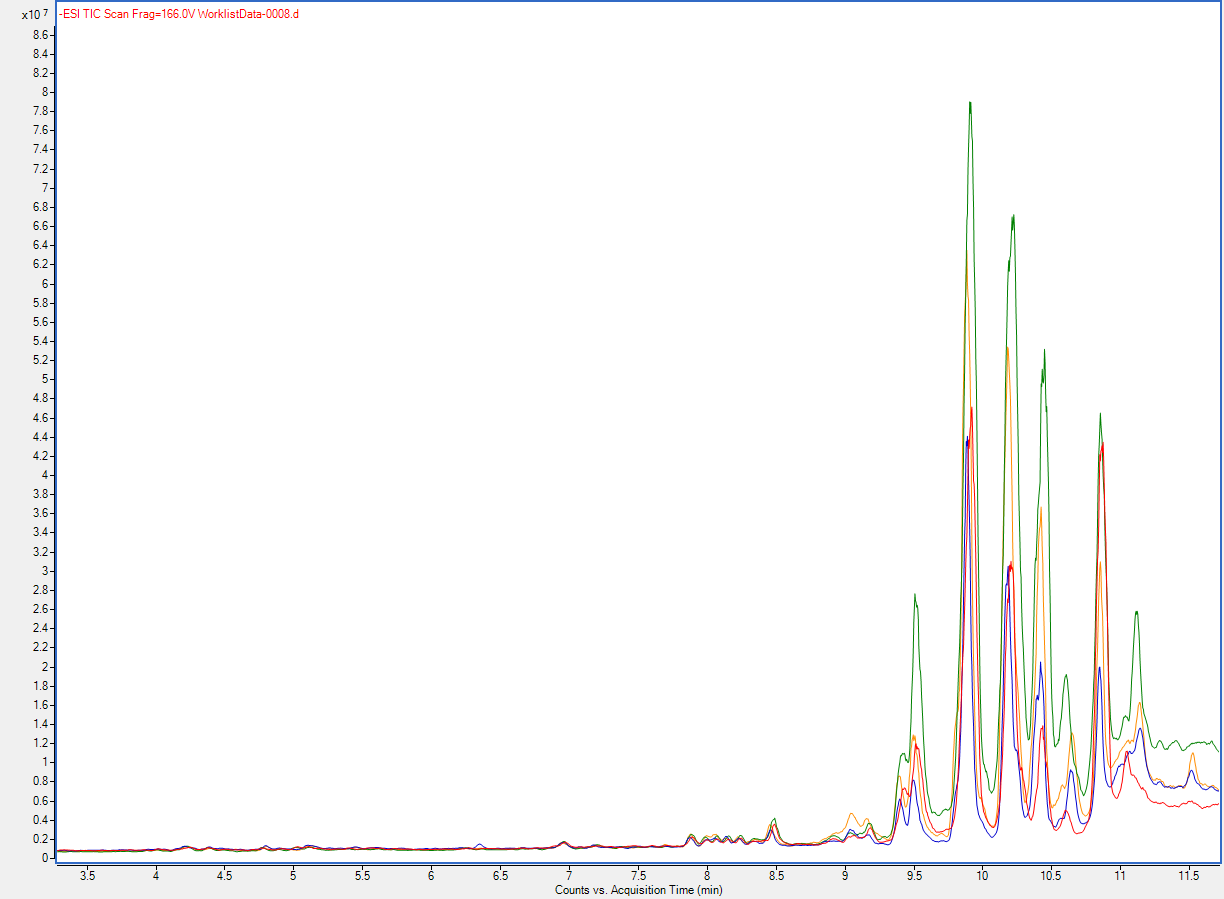 |
| --- |
| Figure S1: Total ion chromatogram response for precursor ion ranging from m/z = 100 – 1300 in chicken serum spiked with 0.5 ng mL^-1^ PFAS for four extraction methodologies: protein precipitation (green), enhanced matrix removal (red), weak anion exchange (orange) and hydrophilic lithophilic balance (blue). |

**References**

[1] Kaiser A-M, Aro R, Kärrman A, Weiss S, Hartmann C, Uhl M, et al. Comparison of extraction methods for per- and polyfluoroalkyl substances (PFAS) in human serum and placenta samples—insights into extractable organic fluorine (EOF). *Anal Bioanal Chem*. 2021; 413 (3): 865-76. <https://doi.org/10.1007/s00216-020-03041-5>

[2] Da Silva BF, Ahmadireskety A, Aristizabal-Henao JJ, Bowden JA. A rapid and simple method to quantify per- and polyfluoroalkyl substances (PFAS) in plasma and serum using 96-well plates. 2020; 7: 101111. <https://doi.org/10.1016/j.mex.2020.101111>

[3] Nakayama SF, Isobe T, Iwai-Shimada M, Kobayashi Y, Nishihama Y, Taniguchi Y, et al. Poly- and perfluoroalkyl substances in maternal serum: Method development and application in Pilot Study of the Japan Environment and Children's Study. *J Chromatogr A*. 2020; 1618: 460933. <https://doi.org/10.1016/j.chroma.2020.460933>

[4] Liu X, Yu Y, Li Y, Zhang H, Ling J, Sun X, et al. Fluorocarbon-bonded magnetic mesoporous microspheres for the analysis of perfluorinated compounds in human serum by high-performance liquid chromatography coupled to tandem mass spectrometry. *Anal Chim Acta*. 2014; 844: 35-43. <https://doi.org/10.1016/j.aca.2014.07.032>

[5] Keller JM, Calafat AM, Kato K, Ellefson ME, Reagen WK, Strynar M, et al. Determination of perfluorinated alkyl acid concentrations in human serum and milk standard reference materials. *Anal Bioanal Chem*. 2010; 397 (2): 439-51. <https://doi.org/10.1007/s00216-009-3222-x>

[6] Yeung LWY, Taniyasu S, Kannan K, Xu DZY, Guruge KS, Lam PKS, et al. An analytical method for the determination of perfluorinated compounds in whole blood using acetonitrile and solid phase extraction methods. *J Chromatogr A*. 2009; 1216 (25): 4950-6. <https://doi.org/10.1016/j.chroma.2009.04.070>

[7] Reagen WK, Ellefson ME, Kannan K, Giesy JP. Comparison of extraction and quantification methods of perfluorinated compounds in human plasma, serum, and whole blood. *Anal Chim Acta*. 2008; 628 (2): 214-21. <https://doi.org/10.1016/j.aca.2008.09.029>

[8] Benskin JP, Bataineh M, Martin JW. Simultaneous characterization of perfluoroalkyl carboxylate, sulfonate, and sulfonamide isomers by liquid chromatography−tandem mass spectrometry. *Anal Chem*. 2007; 79 (17): 6455-64. <https://doi.org/10.1021/ac070802d>

[9] Kärrman A, van Bavel B, Järnberg U, Hardell L, Lindström G. Development of a Solid-Phase Extraction-HPLC/Single Quadrupole MS Method for Quantification of Perfluorochemicals in Whole Blood. *Anal Chem*. 2005; 77 (3): 864-70. <https://doi.org/10.1021/ac049023c>

[10] Kuklenyik Z, Reich JA, Tully JS, Needham LL, Calafat AM. Automated solid-phase extraction and measurement of perfluorinated organic acids and amides in human serum and milk. *Environ Sci Technol*. 2004; 38 (13): 3698-704. <https://doi.org/10.1021/es040332u>

[11] Marchiandi J, Szabo D, Dagnino S, Green MP, Clarke BO. Occurrence and fate of legacy and novel per- and polyfluoroalkyl substances (PFASs) in freshwater after an industrial fire of unknown chemical stockpiles. *Environ Pollut*. 2021: 116839. <https://doi.org/10.1016/j.envpol.2021.116839>

[12] Szabo D, Nuske MR, Lavers JL, Shimeta J, Green MP, Mulder RA, et al. A baseline study of per- and polyfluoroalkyl substances (PFASs) in waterfowl from a remote Australian environment. *Sci Total Environ*. 2022; 812: 152528. <https://doi.org/10.1016/j.scitotenv.2021.152528>
